# Emergence of extensively drug-resistant *Acinetobacter baumannii* complex over 10 years: Nationwide data from the Taiwan Surveillance of Antimicrobial Resistance (TSAR) program

**DOI:** 10.1186/1471-2334-12-200

**Published:** 2012-08-28

**Authors:** Shu-Chen Kuo, Shan-Chwen Chang, Hui-Ying Wang, Jui-Fen Lai, Pei-Chen Chen, Yih-Ru Shiau, I-Wen Huang, Tsai-Ling Yang Lauderdale

**Affiliations:** 1 National Institute of Infectious Diseases and Vaccinology, National Health Research Institutes, No. 35 Keyan Road, Zhunan, Taiwan, 35053; 2 Institute of Clinical Medicine, National Yang-Ming University, School of Medicine, Taipei, Taiwan; 3 Division of Infectious Diseases, Department of Medicine, Taipei Veterans General Hospital, Taipei, Taiwan; 4 Division of Infectious Diseases, Department of Internal Medicine, National Taiwan University Hospital, Taipei, Taiwan; 5 Graduate Institute of Clinical Pharmacy, College of Medicine, National Taiwan University, Taipei, Taiwan

**Keywords:** Extensively drug-resistant, *Acinetobacter baumannii* complex, Antimicrobial resistance

## Abstract

**Background:**

*Acinetobacter baumannii* complex (ABC) has emerged as an important pathogen causing a variety of infections. Longitudinal multicenter surveillance data on ABC from different sources in Taiwan have not been published. Using data from the Taiwan Surveillance of Antimicrobial Resistance (TSAR) conducted biennially, we investigated the secular change in resistance of 1640 ABC from 2002 to 2010 (TSAR period III to VII) to different antimicrobial agents and identified factors associated with imipenem-resistant and extensively drug-resistant ABC (IRABC and XDRABC).

**Methods:**

Isolates were collected by TSAR from the same 26 hospitals located in all 4 regions of Taiwan. Minimum inhibitory concentrations (MIC) were determined by reference broth microdilution method. Isolates nonsusceptible to all tested aminoglycosides, fluoroquinolones, β-lactam, β-lactam/β-lactam inhibitors, and carbapenems were defined as extensively drug-resistant (XDR). Multivariate logistic regression analysis was performed to assess the relationship between predictor variables among patients with resistant ABC and patients with non-resistant ABC.

**Results:**

The prevalence of IRABC increased from 3.4% in 2002 to 58.7% in 2010 (*P* < 0.001; odds ratio [OR], 2.138; 95% confidence interval [CI], 1.947 to 2.347) and that of XDRABC increased from 1.3% in 2002 to 41.0% in 2010 (*P* < 0.001; OR, 1.970; 95% CI, 1.773-2.189). The rates of non-susceptibility to other antimicrobial agents remained high (>55%) over the years with some fluctuations before and after TSAR V (2006) on some agents. Multivariate analysis revealed that recovery from elderly patients, origins other than blood, from ICU settings, or geographic regions are independent factors associated with IRABC and XDRABC. Although the prevalence of XDRABC increased in all four regions of Taiwan over the years, central Taiwan had higher prevalence of XDRABC starting in 2008. Susceptibility to polymyxin remained high (99.8%).

**Conclusions:**

This longitudinal multicenter surveillance program revealed significant increase and nationwide emergence of IRABC and XDRABC in Taiwan over the years. This study also identified factors associated with IRABC and XDRABC to help guide empirical therapy and at-risk groups requiring more intense interventional infection control measures with focused surveillance efforts.

## Background

*Acinetobacter* spp., especially *Acinetobacter baumannii* complex (ABC), has emerged as an important pathogen causing a variety of infections including urinary tract infection, skin and soft tissue infections, and pneumonia and bloodstream infections with high morbidity and mortality [1]. The ability to chronically colonize patients and cause outbreaks which are usually hard to eradicate poses significant challenge to infection control and increases healthcare expenditure [2]. In addition to its intrinsic resistance to many commonly used antibiotics, this troublesome pathogen can gain additional mechanism rapidly in response to new broad-spectrum antibiotics [3,4]. Due to treatment failure, drug-resistant strains have been associated with higher mortality and prolonged hospital stay compared with susceptible ones [5,6].

Carbapenems such as imipenem and meropenem are the last resort of drugs for the treatment of multidrug-resistant pathogens including ABC. However, the incidence of carbapenem resistance in ABC increased steadily in the 2000s [4,7]. In Europe, the MYSTIC program in 2006 revealed a considerable increase in carbapenem resistance rates to 42.5% [8]. Worldwide, the SENTRY program documented an overall increase in imipenem nonsusceptibility from 34.5% in 2006 to 59.8% in 2009 [9]. Imipenem-resistance in Taiwan ranged from 22% in 2000 to 25% in 2005 [10]. Ampicillin/sulbactam, tigecycline, and colistin are possible options for imipenem-resistant ABC but decreasing susceptibility to these agents has also been reported [1]. Surveillance is therefore important in providing useful information for physicians in choosing empirical antibiotics. It also helps to address specific resistant issues within a region to help identify targeted intervention measures [11,12].

Although there have been reports of the high prevalence of drug-resistant ABC in Taiwan [13,14], longitudinal nationwide surveillance data on isolates from different sources in Taiwan have not been published. The Taiwan Surveillance of Antimicrobial Resistance (TSAR) is a nationwide program at the National Health Research Institutes [11] and has been conducted biennially since 1998 [15]. Using data from TSAR, we aimed at detailing the secular change of resistance to various antimicrobial agents in ABC from different sources over 10 years and identify factors associated with imipenem-resistant and extensively drug-resistant ABC (IRABC and XDRABC).

## Methods

### Study period and isolate collection process

The study period spanned from 2002 to 2010 (corresponding to TSAR period III to VII). Bacterial isolates were collected biennially from July to September by the TSAR program from the same 26 hospitals except TSAR V (2006), in which one hospital did not participate. These hospitals comprised 11 medical centers and 15 regional hospitals, and are located in all 4 regions of Taiwan including 7, 8, 8, and 3 in the north, central, south and east region, respectively. The majority of the Taiwan’s population lives in the western part (north, central and south regions) while the eastern part is the least populated region. The collection protocol was similar for all 5 rounds of TSAR as described previously [16,17]. Briefly, each hospital first collected 50 outpatient isolates, 30 adult ICU and 100 non-ICU inpatient isolates, and 20 pediatric isolates. After completion of the above collection, an additional 20 (for TSAR III to V) to 50 (for TSAR VI and VII) isolates from blood and sterile body sites were collected. The isolates were collected sequentially without specifying species. All isolates were stored at −80°C for subsequent testing. The bacterial isolates were recovered from clinical samples taken as part of standard care. The study was approved by the Research Ethics Committee of National Health Research Institutes (EC960205).

### Bacterial isolates and information

For *Acinetobacter* spp., isolates were subcultured to blood agar and McConkey agar plates at our laboratory for purity check and to confirm species identification. Either Vitek I (prior to 2008) or Vitek II (2008 and 2010) GN card was used (bioMérieux, Marcy l'Etoile, France). In addition, conventional biochemical tests including oxidase, Triple Sugar Iron, 42°C, malonate, and hemolysis on sheep blood agar were used to aid in confirmation of the *A. baumannii* complex [18]. The hospital also provided information on specimen source and patient age. For analysis, samples from upper respiratory tract were designated as respiratory origin. Samples from pus/discharge included those from abscesses or wounds.

### Antimicrobial susceptibility

Minimum inhibitory concentrations (MIC) of different agents were determined by reference broth microdilution test following the guidelines of Clinical and Laboratory Standards Institute (CLSI) using custom designed Sensititre panels (Trek Diagnostics, West Essex, England) [19] Amikacin, ampicillin/sulbactam, ceftazidime, cefepime, ciprofloxacin, gentamicin, levofloxacin, imipenem, and piperacillin/tazobactam were tested on all isolates from each study year. Polymyxin B and tigecycline were tested on imipenem-resistant isolates in 2002 and 2004 and on all isolates from 2008–2010. Trimethoprim-sulfamethoxazole was tested on all isolates from 2002, 2004 and 2010. Control strains included *Pseudomonas aeruginosa* ATCC 27853, *Escherichia coli* ATCC 25922 and ATCC 35218. Results were interpreted using the CLSI breakpoints except for colistin and tigecycline, for which the EUCAST breakpoints (for Enterobacteriaceae) were used (http://www.eucast.org/clinical_breakpoints). Isolates nonsusceptible to all the tested aminoglycosides, fluoroquinolones, β-lactam, β-lactam/β-lactam inhibitors, and carbapenems were defined as extensively drug-resistant (XDR) [20,21].This is a modification of the ECDC definition for XDR, which is defined as non-susceptibility to at least one agent in all but two or fewer antimicrobial categories [20].

### Data and statistical analysis

For analysis of susceptibility rates in different year and patient groups, we used the Whonet software [22]. Univariate analysis was done using Student’s *t* test/Mann–Whitney U test, or Fisher’s exact tests as appropriate. Multivariate logistic regression analysis was performed to assess the relationship between predictor variables among patients with resistant ABC and patients with non-resistant ABC. The variables included those identified in the univariate analysis as possibly being associated with resistance rate (*P* < 0.05). For comparison of resistance between regions and specimen types, these variables were entered in the multivariate analysis in a dummy form (resistance rate in northern Taiwan or that of blood samples as the reference group, respectively). For the trend test calculation, a continuous variable (TSAR period III to VII corresponding to 3 to 7, respectively) was used [23]. All analyses were performed with the Statistical Package for the Social Sciences version 18.0 (SPSS, Chicago, IL, USA). A *P* < 0.05 was considered to be statistically significant.

## Results

### Bacterial isolates

Between 2002 and 2010, a total of 1,681 *Acinetobacter* spp. isolates were collected and ABC comprised 97.6% (1,640 isolates). Non-ABC isolates were excluded from subsequent data analysis. Among the 1640 ABC isolates, the mean age of patients was 66.8 ± 19.8 years. Table 1 lists the source breakdown of the isolates for each round of TSAR. Isolates were mostly recovered from respiratory samples (822 isolates, 50.1%), followed by blood (244, 14.9%), pus/discharge (219, 13.4%) and urine (216, 13.2%). Seven hundred and thirty-five (44.8%) were from medical centers and 600 (36.6%) were from ICU. Isolates from central Taiwan comprised the largest proportion (627, 38.2%) (Table 1).

**Table 1:** **Source breakdown of* Acinetobacter baumannii *complex from the Taiwan Surveillance of Antimicrobial Resistance (TSAR) by year**

**Strata**	**TSAR III (2002)**		**TSAR IV (2004)**		**TSAR V (2006)**		**TSAR VI (2008)**		**TSAR VII (2010)**		**TSAR III-VII Combined**		***P***^***a***^
	**N**	**%**	**N**	**%**	**N**	**%**	**N**	**%**	**N**	**%**	**N**	**%**	
Hospital type													
Medical centers (11)	162	54.4	124	47.9	126	41.4	172	41.1	151	41.8	735	44.8	0.002
Regional hospitals (15)	136	45.6	135	52.1	178	58.6	246	58.9	210	58.2	905	55.2	0.002
Region													
North (7)	90	30.2	70	27.0	76	25.0	118	28.2	118	32.7	472	28.8	0.2354
Central (8)	94	31.5	91	35.1	109	35.9	189	45.2	144	39.9	627	38.2	0.0024
South (8)	77	25.8	57	22.0	76	25.0	65	15.6	68	18.8	343	20.9	0.0032
East (3)	37	12.4	41	15.8	43	14.1	46	11.0	31	8.6	198	12.1	0.0194
Patient location													
ICU	100	33.6	105	40.5	103	33.9	157	37.6	135	37.4	600	36.6	0.3848
Non-ICU	174	58.4	138	53.3	165	54.3	227	54.3	201	55.7	905	55.2	0.7578
OPD/ER	24	8.1	16	6.2	36	11.8	34	8.1	25	6.9	135	8.2	0.1104
Specimen type													
Respiratory	157	52.7	136	52.5	137	45.1	208	49.8	184	51.0	822	50.1	0.3294
Blood	33	11.1	28	10.8	34	11.2	84	20.1	65	18.0	244	14.9	0.0002
Pus/discharge	49	16.4	40	15.4	56	18.4	40	9.6	34	9.4	219	13.4	0.0005
Urine	34	11.4	30	11.6	50	16.4	57	13.6	45	12.5	216	13.2	0.332
Others	25	8.4	25	9.7	27	8.9	29	6.9	33	9.1	139	8.5	0.7321
Total	298		259		304		418		361		1640		

^a^The differences of hospital types, regions, patient locations, and specimen types among TSAR III to VII were compared using Pearson's chi-squared test.

### Changes in non-susceptibility to different antimicrobial agents over the years

**Figure 1:**
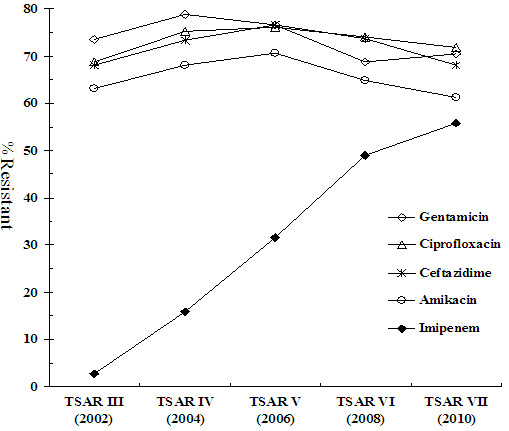
**Rapid increase of imipenem-resistance in* Acinetobacter baumannii *complex (OR 2.277; 95% CI, 2.054-2.523;*P* < 0.001) in Taiwan based on the Taiwan Surveillance of Antimicrobial Resistance (TSAR) data.** The secular trends of resistance to 4 other agents are shown for comparison.

**Figure 2:**
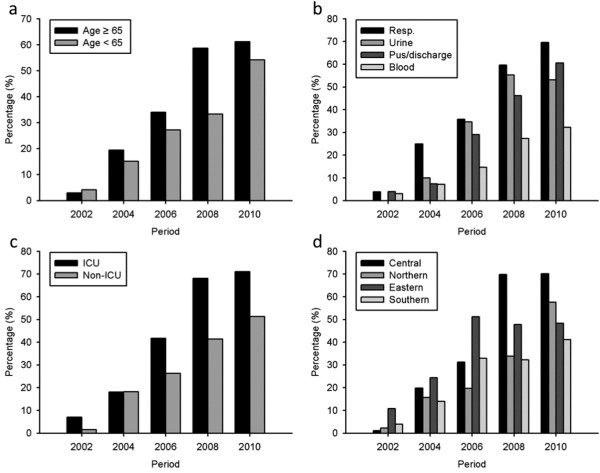
**Secular trend of imipenem-resistance rate in* Acinetobacter baumannii *complex recovered from 2002 to 2010 in Taiwan.** Differences in resistance rates were stratified by (**a**) patient age, (**b**) sample origins, (**c**) healthcare settings, and (**d**) geographic regions, based on the Taiwan Surveillance of Antimicrobial Resistance (TSAR) data.

Rates of non-susceptibility to different antimicrobial agents between 2002 and 2010 are shown in Table 2. The secular trend of non-susceptibility to amikacin, ceftazidime, levofloxacin, and imipenem over the 10 years is also shown in Figure 1 to highlight the sharp increase of carbapenem (imipenem) non-susceptibility from 3.4% in 2002 to 58.7% in 2010 (*P* < 0.001; odds ratio [OR], 2.138; 95% confidence interval [CI], 1.947 to 2.347). The increased carbapenem-resistance was observed in isolates from elderly as well as non-elderly patients, and in those from different specimen types, ICU and non-ICU patients, and throughout different regions of Taiwan (Figure 2).

**Table 2:** **Secular trend of antimicrobial non-susceptibility in* Acinetobacter baumannii *complex from the 2002 to 2010 Taiwan Surveillance of Antimicrobial Resistance (TSAR) program**

**Antimicrobial agents**^**a**^	**Resistance rate (%) by year**					**2002 to 2006**^**b**^			**2006 to 2010**		
	**2002 (298)**	**2004 (259)**	**2006 (304)**	**2008 (418)**	**2010 (361)**	***P***	**OR**	**95% CI**	***P***	**OR**	**95% CI**
Amikacin	63.1	68	70.7	66.5	64.3	0.046	1.19	1.003-1.411	0.081	0.865	0.735-1.018
Amp/Sulb	57.4	64.1	57.6	61.5	59.6	0.971	1.003	0.852-1.180	0.637	1.038	0.889-1.212
Cefepime	64.1	70.7	74	71.3	60.9	0.008	1.265	1.062-1.507	<0.001	0.731	0.619-0.864
Ceftazidime	68.1	73.4	76.6	74.6	70.4	0.019	1.241	1.036-1.486	0.063	0.848	0.712-1.009
Ciprofloxacin	68.8	75.3	76	75.4	75.3	0.047	1.202	1.002-1.441	0.853	0.983	0.824-1.174
Gentamicin	73.5	78.8	76.6	70.3	72.9	0.367	1.09	0.904-1.315	0.311	0.915	0.770-1.087
Imipenem	3.4	18.1	31.6	51.4	58.7	<0.001	3.043	2.348-3.944	<0.001	1.732	1.478-2.029
Levofloxacin	66.1	72.6	74.3	73.9	71.2	0.027	1.222	1.023-1.458	0.349	0.921	0.775-1.094
Pip/Tazo	62.8	76.8	75.3	74.6	68.4	0.001	1.363	1.139-1.631	0.041	0.835	0.703-0.992
TMP/SMX	73.8	75.7	NT^c^	NT	71.5	ND^d^			ND		

^a^Amp/Sulb, Ampicillin/sulbactam; Pip/Tazo, Piperacillin/tazobactam; TMP/SMX, trimethoprim/sulfamethoxazole.

^b^OR, odds ratio; CI, confidence interval.

^c^NT, not tested.

^d^ND, not done.

The rates of non-susceptibility to other antimicrobial agents remained high (>55%) over the years although fluctuations were observed before and after TSAR V (2006) on some agents (Table 2 & Figure 1); non-susceptibility to amikacin, cefepime, ceftazidime, levofloxacin, and piperacillin/tazobactam increased significantly before 2006 (TSAR V), then stayed at a plateau and even declined thereafter (Table 2). In contrast, the rates of non-susceptibility to ampicillin/sulbactam (range 56.1% - 64.1%) fluctuated over 10 years without significant change (*P* = 0.775; OR, 1.010; 95% CI, 0.942 to 1.084), while that of gentamicin decreased slightly since 2004 (*P* = 0.034; OR, 0.883; 95% CI, 0.787 to 0.991). The overall rate of susceptibility to tigecycline (MIC < = 2 mg/L) and polymyxin B was 97.7% and 99.8%, respectively in 1,160 tested isolates and 98.1% and 100%, respectively in IRABC.

### Factors associated with emergence of imipenem-resistant *A. baumannii* complex (IRABC)

Table 3 presents the factors associated with the emergence of IRABC. Isolates from elderly patients (> 65 years old), respiratory tract origin, ICU settings, or central Taiwan were significantly associated with imipenem resistance whereas rates of imipenem-resistance were lower in isolates from blood or pus/discharge, medical centers, southern or northern Taiwan (Figure 2). Multivariate analysis revealed recovery from elderly patients, origins other than blood, ICU settings, geographic region, and latter collection year remained independent factors (Table 3).

**Table 3:** **Factors associated with imipenem-resistant* Acinetobacter baumannii *complex (IRABC) in Taiwan**

**Characteristic**	**Non-IRABC**	**IRABC**	***P***^**a**^	**OR**^**b**^	**95% CI**^**b**^	***P***^**b**^
Number	1060	580				
Mean age ± SD^c^	65.0 ± 20.2	70.0 ± 18.8	<0.001			
Age 65 and older^c^	629 (61.1)	401 (71.9)	<0.001	1.464	1.122-1.912	0.005
Specimen types						
Respiratory tracts	481 (45.4)	341 (58.8)	<0.001	2.597	1.888-3.570	<0.001
Blood	192 (18.1)	52 (9.0)	<0.001			Reference
Pus/discharge	160 (15.1)	59 (10.2)	0.005	1.934	1.240-3.018	0.004
Urine	140 (13.2)	76 (13.1)	>0.99	2.138	1.401-3.263	<0.001
Healthcare settings						
Medical centers	507 (47.8)	228 (39.3)	0.001	0.814	0.631-1.049	0.112
ICU Settings	328 (30.9)	272 (46.9)	<0.001	1.919	1.482-2.486	<0.001
Geographic regions						
Northern	336 (31.7)	136 (23.4)	<0.001			Reference
Central	341 (32.2)	286 (49.3)	<0.001	2.43	1.791-3.296	<0.001
Southern	258 (24.3)	85 (14.7)	<0.001	1.178	0.812-1.710	0.388
Eastern	125 (11.8)	73 (12.6)	0.635	2.24	1.489-3.370	<0.001
TSAR period^d^				2.277	2.054-2.523	<0.001

^a^*P* value by chi-square test.

^b^*P* value by multivariate analysis; OR, odds ratio; CI, confidence interval.

^c^The patient ages for 50 patients were unknown.

^d^Taiwan Surveillance of Antimicrobial Resistance (TSAR) III (2002) to VII (2010).

### Emergence of extensively drug-resistant *A. baumannii* complex (XDRABC)

**Figure 3:**
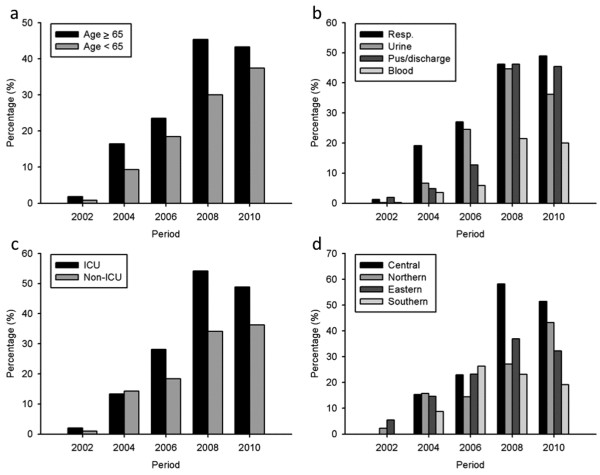
**Secular trend of extensively drug-resistance rate in* Acinetobacter baumannii *complex recovered from 2002 to 2010 in Taiwan.** Differences in resistance rates were stratified by (**a**) patient age (**b**) sample origins (**c**) healthcare settings (**d**) geographic regions based on the Taiwan Surveillance of Antimicrobial Resistance (TSAR) data.

The prevalence of XDRABC increased significantly from 1.3% in 2002 to 41.0% in 2010, respectively (*P* <0.001; OR, 1.970; 95% CI, 1.773-2.189), with an overall prevalence of 26.1% (428 isolates) over the 10 years. The increased XDRABC also occurred in isolates from elderly and non-elderly patients both, and in those from different specimen types, ICU as well as non-ICU patients, while central region saw a sharp increase of XDRABC in 2008–2010 (Figure 3). Ten (2.3%) of the XDRABC had tigecycline > 2 mg/L and all were susceptible to polymyxin B. Trimethoprim-sulfamethoxazole was tested on 187 XDRABC isolates and all but 4 were resistant.

### Factors associated with emergence of XDRABC

Factors associated with XDRABC strains included recovery from elderly patients (> 65 years old), respiratory tract origin, ICU settings, or central Taiwan (Table 4). The extensively drug-resistant rates over 10 years stratified by these independent factors are shown in Figure 3. Isolates from blood or pus/discharge, medical center, southern or northern Taiwan were less likely to be XDR. The independent factors associated with XDRABC included recovery from elderly patients, origins other than blood, ICU settings, geographic region, and latter collection year (Table 4).

**Table 4:** **Factors associated with extensively drug-resistant* Acinetobacter baumannii *complex (XDRABC) in Taiwan**

**Characteristic**	**Non-XDRABC**	**XDRABC**	***P***^**a**^	**OR**^**b**^	**95% CI**^**b**^	***P***^**b**^
Number	1212	428				
Mean age ± SD^c^	65.4 ± 20.2	70.6 ± 18.1	<0.001			
Age 65 and older^c^	733 (71.2)	297 (28.8)	<0.001	1.39	1.052-1.836	0.021
Specimens						
Respiratory tracts	571 (47.1)	251 (58.6)	<0.001	2.102	1.507-2.932	<0.001
Blood	210 (17.3)	34 (7.9)	<0.001			Reference
Pus/discharge	176 (14.5)	43 (10.0)	0.02	1.598	0.996-2.568	0.052
Urine	160 (13.2)	56 (13.1)	>0.99	1.759	1.130-2.739	0.012
Healthcare settings						
Medical centers	570 (47.0)	165 (38.6)	0.003	0.804	0.617-1.046	0.104
ICU settings	404 (33.3)	196 (45.8)	<0.001	1.468	1.126-1.915	0.005
Geographic regions						
Northern	365 (30.1)	107 (25.0)	0.047			Reference
Central	404 (33.3)	223 (52.1)	<0.001	1.965	1.466-2.671	<0.001
Southern	290 (23.9)	53 (12.4)	<0.001	0.814	0.544-1.216	0.314
Eastern	153 (12.6)	45 (10.5)	0.263	1.303	0.847-2.006	0.229
TSAR period^d^				1.97	1.773-2.189	<0.001

^a^*P* value by chi-square test.

^b^*P* value by multivariate analysis; OR, odds ratio; CI, confidence interval.

^c^The patient ages of 50 patients were unknown.

^d^Taiwan Surveillance of Antimicrobial Resistance (TSAR) III (2002) to VII (2010).

## Discussion

This nationwide longitudinal study of 1,640 ABC over 10 years revealed the continuous increase of non-susceptibility to imipenem and emergence of XDRABC. For most other antibiotics, resistance rate increased from 2002 to 2006 but ceased to increase or even decreased thereafter. The prevalence of IRABC and XDRABC was independently affected by age of patients, specimen types, healthcare settings, and geographic regions.

Resistance to imipenem, which is often accompanied with resistance to multiple other agents, has increased in all parts of the world, ranging from 14.1% in Europe to 39.4% in Latin America in 2004–2006 [24], and from 34.5% in 2006 to 59.8% in 2009 overall worldwide [9]. Our study revealed the rapid increase in the prevalence of imipenem-resistance over 10 years in Taiwan, from 3.4% in 2002 to 58.7% in 2010. The imipenem-nonsusceptible rate of 62.6% in isolates from Asia-Pacific in 2009 reported by the SENTRY study was comparable to our result [9].

Although XDRABC in Taiwan has been observed in other pilot studies [10,14], this is the first study addressing the emergence of XDRABC in Taiwan over a long period. However, the variety of definition regarding XDR precluded the comparison of our data with previous ones. The definition of XDR in our study was in accordance to that of Tan et al. [21] and approached the consensus of European Centre for Disease Prevention and Control [20] except that not all isolates were tested for trimethoprim-sulfamethoxazole. However, almost all of XDRABC tested showed resistance to it.

Prolonged hospitalization, ICU stay, invasive medical procedures, and prior broad-spectrum antibiotic use, especially carbapenem, third-generation cephalosporins, and fluoroquinolones, have been shown to be risk factors for acquisition of IRABC [4,7,25]. These factors, as well as clonal dissemination [26,27], likely all contributed to the increased prevalence of IRABC and XDRABC in Taiwan over the years. A recent study found carbapenem use in Taiwan hospitals increased by 86% between 2003 and 2008, which was significantly associated with increased healthcare associated IRABC infections [28]. The same study also found that the largest increase in carbapenem use occurred in the central region, the area we found the highest prevalence of IRABC and XDRABC. Strain variability and differences in oxacillinase distribution [26,27] may also account for regional differences and the changes in resistance after 2006, and remain to be investigated.

Based on the independent associated factors we identified, the prevalence of imipenem-resistance and XDRABC varied greatly. To our knowledge, variations in resistance profiles among ABC from different sample origins have rarely been addressed before. Whether the lower resistance of isolates from blood compared to other sample origins resulted from different clonality and/or impaired virulence in the cost of resistance [29] remained to be elucidated. Varied prevalence among different areas and healthcare settings (ICU) in Asia has been observed [10]. The insignificant difference between medical centers and regional hospitals may have resulted from frequent transportation of patients and high density of hospitals in Taiwan. The advanced age may imply the underlying conditions of the patients. Higher prevalence of IRABC and XDRABC in ICU and regional variations within Taiwan likely reflect differences in patient populations and antibiotic use [28,30].

The rate of resistance to polymyxin B in our study was only 0.2%, which is similar to that found in isolates from the Asia-Western Pacific region between 2006 and 2009 [9]. In contrast, one study in Korea reported a resistance rate of 18.1% [31]. As for tigecycline, 0.2% of IRABC isolates had MIC > 2 mg/L in Asia region [32] whereas our study found it to be 1.9% in Taiwan. Susceptibility to polymyxin remained high even for the XDRABC isolates in our study. However, in a pilot study by Jean et al., [14], the authors reported higher rate of resistance to colistin and tigecycline in Taiwan. The source of isolates differed in the two studies. Ours were from different patient groups of both teaching and regional hospitals while theirs were from ICUs of ten major teaching hospitals. The underlying conditions, disease severity, and amount of colistin and tigecycline used would be different. However, both studies revealed that compared to other antibiotics, tigecycline and polymyxin B (or its less toxic derivatives, colistin) remained an in vitro effective antimicrobial agent for the treatment of IRABC or XDRABC in Taiwan.

## Conclusion

This longitudinal multicenter surveillance program revealed significant increase and nationwide emergence of IRABC and XDRABC in Taiwan despite that non-susceptibility to other antibiotics remained stable or declined over the past 6 years. This study also identified factors associated with their resistance to help guide empirical therapy and at-risk groups requiring more intense interventional infection control measures with focused surveillance efforts.

## Abbreviations

IRABC, Imipenem-Resistant *Acinetobacter baumannii* Complex; XDRABC, Extensively Drug-Resistant *Acinetobacter baumannii* Complex.

## Competing interests

The authors declare that they have no competing interests.

## Authors' contributions

SCK performed data analysis and interpretation, and drafted the manuscript.

SCC participated in the design and data interpretation of the study and helped to finalize the manuscript.

HYW, JFL, PCC, YRS and IWH carried out the laboratory assays and participated in data analysis.

TLL designed and supervised the study, participated in data analysis and interpretation, and finalized the manuscript.

All authors read and approved the final version of the manuscript.

## Pre-publication history

The pre-publication history for this paper can be accessed here:

http://www.biomedcentral.com/1471-2334/12/200/prepub

## References

[B1] Peleg AY Seifert H Paterson DL Acinetobacter baumannii: emergence of a successful pathogen Clin Microbiol Rev 2008 21 538 582 10.1128/CMR.00058-07 18625687 PMC2493088

[B2] Dijkshoorn L Nemec A Seifert H An increasing threat in hospitals: multidrug-resistant Acinetobacter baumannii Nat Rev Microbiol 2007 5 939 951 10.1038/nrmicro1789 18007677

[B3] Neonakis IK Spandidos DA Petinaki E Confronting multidrug-resistant Acinetobacter baumannii: a review Int J Antimicrob Agents 2011 37 102 109 10.1016/j.ijantimicag.2010.10.014 21130607

[B4] Perez F Hujer AM Hujer KM Decker BK Rather PN Bonomo RA Global challenge of multidrug-resistant Acinetobacter baumannii Antimicrob Agents Chemother 2007 51 3471 3484 10.1128/AAC.01464-06 17646423 PMC2043292

[B5] Kwon KT Oh WS Song JH Chang HH Jung SI Kim SW Ryu SY Heo ST Jung DS Rhee JY Impact of imipenem resistance on mortality in patients with Acinetobacter bacteraemia J Antimicrob Chemother 2007 59 525 530 10.1093/jac/dkl499 17213265

[B6] Sunenshine RH Wright M-O Maragakis LL Harris AD Song X Hebden J Cosgrove SE Anderson A Carnell J Jernigan DB Multidrug-resistant Acinetobacter infection mortality rate and length of hospitalization Emerg Infect Dis 2007 13 97 103 10.3201/eid1301.060716 17370521 PMC2725827

[B7] Peleg AY Paterson DL Multidrug-resistant Acinetobacter: a threat to the antibiotic era Intern Med J 2006 36 479 482 10.1111/j.1445-5994.2006.01130.x 16866649

[B8] Turner PJ Meropenem activity against European isolates: report on the MYSTIC (Meropenem Yearly Susceptibility Test Information Collection) 2006 results Diagn Microbiol Infect Dis 2008 60 185 192 10.1016/j.diagmicrobio.2007.09.006 17945455

[B9] Gales AC Jones RN Sader HS Contemporary activity of colistin and polymyxin B against a worldwide collection of Gram-negative pathogens: results from the SENTRY Antimicrobial Surveillance Program (2006–09) J Antimicrob Chemother 2011 66 2070 2074 10.1093/jac/dkr239 21715434

[B10] Jean S-S Hsueh P-R High burden of antimicrobial resistance in Asia Int J Antimicrob Agents 2011 37 291 295 10.1016/j.ijantimicag.2011.01.009 21382699

[B11] Lauderdale TL Clifford McDonald L Shiau YR Chen PC Wang HY Lai JF Ho M The status of antimicrobial resistance in Taiwan among gram-negative pathogens: the Taiwan surveillance of antimicrobial resistance (TSAR) program, 2000 Diagn Microbiol Infect Dis 2004 48 211 219 10.1016/j.diagmicrobio.2003.10.005 15023432

[B12] White AR Surveillance BWPoR The British Society for Antimicrobial Chemotherapy Resistance Surveillance Project: a successful collaborative model J Antimicrob Chemother 2008 62 2 ii3 ii14 10.1093/jac/dkn348 18819978

[B13] Tseng SH Lee CM Lin TY Chang SC Chang FY Emergence and spread of multi-drug resistant organisms: think globally and act locally J Microbiol Immunol Infect 2011 44 157 165 10.1016/j.jmii.2011.03.001 21524608

[B14] Jean S-S Hsueh P-R Lee W-S Chang H-T Chou M-Y Chen I-S Wang J-H Lin C-F Shyr J-M Ko W-C Nationwide surveillance of antimicrobial resistance among non-fermentative Gram-negative bacteria in Intensive Care Units in Taiwan: SMART programme data 2005 Int J Antimicrob Agents 2009 33 266 271 10.1016/j.ijantimicag.2008.08.026 19091522

[B15] Ho M McDonald LC Lauderdale TL Yeh LL Chen PC Shiau YR Surveillance of antibiotic resistance in Taiwan, 1998 J Microbiol Immunol Infect 1999 32 239 249 10650488

[B16] Chen FJ Huang IW Wang CH Chen PC Wang HY Lai JF Shiau YR Lauderdale TL mecA-positive Staphylococcus aureus with low-level oxacillin MIC in Taiwan J Clin Microbiol 2012 50 1679 1683 10.1128/JCM.06711-11 22378906 PMC3347131

[B17] Wu H Wang JT Shiau YR Wang HY Lauderdale TL Chang SC A multicenter surveillance of antimicrobial resistance on Stenotrophomonas maltophilia in Taiwan J Microbiol Immunol Infect 2012 45 120 126 10.1016/j.jmii.2011.09.028 22154599

[B18] Schreckenberger PC von Graevenitz A Murray PR, Baron EJ, Pfaller MA, Tenover FC, Yolken RH Acinetobacter, Achromobacter, Alcaligenes, Moraxella, and other nonfermentative Gram-negative rods. p. 539–560 Manual of Clinical Microbiology 1999 7 American Society for Microbiology, Washington, D.C.

[B19] Clinical and Laboratory Standards Institute (CLSI) Performance Standards for Antimicrobial Susceptibility testing; Twenty-First Information Supplement 2011 CLSI document M100-S21, CLSI, Wayne, PA

[B20] Magiorakos AP Srinivasan A Carey RB Carmeli Y Falagas ME Giske CG Harbarth S Hindler JF Kahlmeter G Olsson-Liljequist B Multidrug-resistant, extensively drug-resistant and pandrug-resistant bacteria: an international expert proposal for interim standard definitions for acquired resistance Clin Microbiol Infect 2012 18 268 281 10.1111/j.1469-0691.2011.03570.x 21793988

[B21] Tan TY Lim TP Lee WH Sasikala S Hsu LY Kwa AL In vitro antibiotic synergy in extensively drug-resistant Acinetobacter baumannii: the effect of testing by time-kill, checkerboard, and Etest methods Antimicrob Agents Chemother 2011 55 436 438 10.1128/AAC.00850-10 20956606 PMC3019682

[B22] Stelling JM O'Brien TF Surveillance of antimicrobial resistance: the WHONET program Clin Infect Dis 1997 24 Suppl 1 S157 168 8994799 10.1093/clinids/24.supplement_1.s157

[B23] Scarmeas N Luchsinger JA Schupf N Brickman AM Cosentino S Tang MX Stern Y Physical activity, diet, and risk of Alzheimer disease JAMA 2009 302 627 637 10.1001/jama.2009.1144 19671904 PMC2765045

[B24] Reinert RR Low DE Rossi F Zhang X Wattal C Dowzicky MJ Antimicrobial susceptibility among organisms from the Asia/Pacific Rim, Europe and Latin and North America collected as part of TEST and the in vitro activity of tigecycline J Antimicrob Chemother 2007 60 1018 1029 10.1093/jac/dkm310 17855724

[B25] Falagas ME Kopterides P Risk factors for the isolation of multi-drug-resistant Acinetobacter baumannii and Pseudomonas aeruginosa: a systematic review of the literature J Hosp Infect 2006 64 7 15 10.1016/j.jhin.2006.04.015 16822583

[B26] Lin MF Kuo HY Yeh HW Yang CM Sung CH Tu CC Huang ML Liou ML Emergence and dissemination of bla(OXA-23)-carrying imipenem-resistant Acinetobacter sp. in a regional hospital in Taiwan J Microbiol Immunol Infect 2011 44 39 44 10.1016/j.jmii.2011.01.008 21531351

[B27] Lu PL Doumith M Livermore DM Chen TP Woodford N Diversity of carbapenem resistance mechanisms in Acinetobacter baumannii from a Taiwan hospital: spread of plasmid-borne OXA-72 carbapenemase J Antimicrob Chemother 2009 63 641 647 10.1093/jac/dkn553 19182237

[B28] Su CH Wang JT Hsiung CA Chien LJ Chi CL Yu HT Chang FY Chang SC Increase of carbapenem-resistant Acinetobacter baumannii infection in acute care hospitals in Taiwan: association with hospital antimicrobial usage PLoS One 2012 7 e37788 10.1371/journal.pone.0037788 22629456 PMC3357347

[B29] Smani Y Lopez-Rojas R Dominguez-Herrera J Docobo-Perez F Marti S Vila J Pachon J In vitro and in vivo reduced fitness and virulence in ciprofloxacin-resistant Acinetobacter baumannii Clin Microbiol Infect 2012 18 E1 E4 10.1111/j.1469-0691.2011.03695.x 22084991

[B30] Cao J Song W Gu B Mei YN Tang JP Meng L Yang CQ Wang H Zhou H Correlation between carbapenem consumption and antimicrobial resistance rates of Acinetobacter baumannii in a university-affiliated hospital in China J Clin Pharmacol 2012 Feb 02 [Epub ahead of print] 10.1177/0091270011435988 23400749

[B31] Ko KS Suh JY Kwon KT Jung S-I Park K-H Kang CI Chung DR Peck KR Song J-H High rates of resistance to colistin and polymyxin B in subgroups of Acinetobacter baumannii isolates from Korea J Antimicrob Chemother 2007 60 1163 1167 10.1093/jac/dkm305 17761499

[B32] Farrell DJ Turnidge JD Bell J Sader HS Jones RN The in vitro evaluation of tigecycline tested against pathogens isolated in eight countries in the Asia-Western Pacific region (2008) J Infect 2010 60 440 451 10.1016/j.jinf.2010.03.024 20361999

